# Quantitative attribution of spatio-temporal pattern of pm2.5 concentration based on geodetector and GWR model: Evidence from China’s three major urban agglomerations

**DOI:** 10.1371/journal.pone.0326241

**Published:** 2025-07-07

**Authors:** Zeduo Zou, Xiuyan Zhao, Shuyuan Liu, Xiaodie Yuan, Chunshan Zhou

**Affiliations:** School of Geography and Planning, Sun Yat-sen University, Guangzhou, China; Lanzhou Jiaotong University, CHINA

## Abstract

Clarifying the spatio-temporal evolution of PM2.5 concentration law and its driving mechanism is crucial for the prevention and control of air pollution in urban agglomerations, also helping promote their high-quality development. Based on remote sensing and statistics of urban agglomerations in China’s Beijing-Tianjin-Hebei (BTH), Yangtze River Delta (YRD), and Pearl River Delta (PRD) from 2005 to 2020, the paper analyses the evolution characteristics of the pollution concentration pattern and identifies the influencing factors through spatial analysis method combining the geodetector and geographically weighted regression (GWR) model. As the results show, during the study period: (1) Temporal Trends: annual PM2.5 concentrations exhibited significant declines, with BTH decreasing from 1004.71 μg/m^3^ (2006) to 528 μg/m^3^ (2020), YRD from 1434.81 μg/m^3^ (2008) to 621 μg/m^3^, and PRD from 405.02 μg/m^3^ (2007) to 292 μg/m^3^. The ranking remained YRD > BTH > PRD throughout the study period. (2) Spatial Heterogeneity: Spatial clustering (Moran’s I: 0.286–0.729, p < 0.05) dominated all regions. BTH showed a “high-south” pattern (e.g., Xingtai: 78.3 μg/m^3^ vs. Qinhuangdao: 34.2 μg/m^3^), YRD displayed “high-northwest” characteristics (Hefei: 68.5 μg/m^3^ vs. Ningbo: 42.1 μg/m^3^), while PRD exhibited a west-east gradient (Foshan: 49.8 μg/m^3^ vs. Shenzhen: 25.6 μg/m^3^). (3) The evolution of PM2.5 concentration in three urban agglomerations is generally positive autocorrelative aggregative distribution, and aggregation types include “high-high”, “low-low” and “high-low”. (4) The measurement of geographical detector indicates the differentiation of PM2.5 concentration is affected by both natural geography and socio-economic factors, and the former ones have stronger driving forces. (5) The measurement of GWR model indicates temperature, precipitation, vegetation coverage, urban expansion, industrial structure, and energy efficiency are main influencing factors of PM2.5 concentration pattern, and the degree of influence of these factors is different.

## 1. Introduction

Air pollution is closely related to public health and has emerged as a significant environmental challenge on a global scale [1]. The rapid processes of industrialization and urbanization in China have led to increasingly pronounced environmental pollution issues [2], with air pollution being particularly severe. PM2.5, a major pollutants in the atmosphere, poses significant health risks to humans. Relevant studies have made known that exposure to PM2.5 is associated with a range of respiratory and cardiovascular diseases, as well as detrimental effects on the immune system [3,4]. The high concentration of PM2.5 in the air is the main component of haze [5]. In recent years, cities in China, especially megacities and urban agglomerations, have frequently encountered haze weather. Despite the implementation of laws and regulations, as well as improvements in air quality through adjustments to industrial and energy structures, PM2.5 remains a significant focus and challenge in the control of air pollution in China [6]. As China transitions to a stage of high-quality development, the major task is to accelerate the formation of the green development model. Given the significant socio-economic status of megacities and urban agglomerations, the interplay between development and environmental issues in these regions warrants thorough examination.

Research on PM2.5 has achieved fruitful results. The United States, Spain, and Canada were the first to start studies on PM2.5 pollution standards [7]; in 2012, China issued the “Ambient Air Quality Standard” (GB3095–2012), which includes PM2.5 intothe new category of air quality for the first time, marking the integration with international standards [8,9]. In terms of PM2.5 characteristics, its chemical characteristics [10], geographical agglomeration [11], and spatial heterogeneity [12] have been detected. In terms of influencing factors of PM2.5 concentration, socio-economic factors [13–15] containing per capita GDP, urbanisation rate, population density, urban expansion, industrial structure, and energy consumption have been thoroughly examined; natural geographical factors [16–19] containing temperature, precipitation, wind speed, relative humidity, sunshine hours, and vegetation coverage have also been sufficiently analysed. The methods of these studies involve geographically weighted regression (GWR) [20], Bayesian GWR [21], two-stage distributed lag model [22], geodetector analysis [23], grey relational model [24], principal component analysis [25], spatial econometrics, and mixed regression [26], etc.; the diverse research scales involve countrywide [27], provinces [28], prefectures [29], and urban agglomerations [30–32]. However, the empirical studies mentioned above are generally aimed at single regions, withlittle existing literature to compare the PM2.5 pollution problems across different urban agglomerations.

Urban agglomerations are the inevitable products of the development of urbanisation and industrialisation industrialization, serving as territorial complexes to realise the integration of cities [33,34]. Large urban agglomerations are often function as hubs and gateways that represent the country’s participation in the world urban system and the international labour division system, acting as a region’s growth pole – the most dynamic and competitive areas [35]. For China, urban agglomerations are meant to be supporting areas for formation of harmony between human and nature while promoting sustainable. The Beijing-Tianjin-Hebei (BTH), Yangtze River Delta (YRD) and Pearl River Delta (PRD) urban agglomerations are the three major economic cores of the country, but are accompanied by serious air pollution as well. Their PM2.5 pollution problems have become the focus of the authorities, society, and academia [36,37]. Based on remote sensing data of PM2.5 from 2005 to 2020 and multi-source geographic and socio-economic data, this study focuses on the three major urban agglomerations in China: Beijing-Tianjin-Hebei (BTH), the Yangtze River Delta (YRD), and the Pearl River Delta (PRD) The objectives of the study are as follows:

(1) To quantify the annual average PM2.5 concentrations in the three major urban agglomerations and their internal ‘big city groups’ (e.g., Beijing, Shanghai, Shenzhen, etc.) through remote sensing data extraction and spatial statistics; (2) To analyse the temporal and spatial correlation characteristics of the pollution by using exploratory spatial data analysis (ESDA) and spatial autocorrelation, and to investigate the spatial and temporal evolution of PM2.5; (3) To integrate geodetector with geographically weighted regression (GWR) models to measure the natural geographic and socio-economic driving factors of PM2.5, constructing a “global-local” dual-dimensional analysis framework. The main innovations and advantages of this paper are: first, the innovation of multi-dimensional coupling analysis method between geodetector and GWR model. Breaking through the limitations of the traditional single model, the geodetic detector is deeply integrated with the geographically weighted regression (GWR) model, and a two-dimensional analysis framework of ‘global driver diagnosis-local spatial effect deconstruction’ is constructed to elucidate the composite mechanism of natural constraints and socio-economic activities on the PM2.5 concentration pattern. The advantage of this method is that it can directly quantify the spatiotemporal relationship between independent variables and dependent variables. By observing the spatial and temporal non-stationarity of the influencing factors within urban agglomerations, the findings of this research yield a more detailed and scientifically robust understanding of the subject matter. Second, the innovation of the analysis paradigm of ‘scale hierarchy - spatio-temporal correlation’ within city clusters. Breaking through the generality of existing studies in analysing the urban agglomerations as a whole, the study innovatively proposes a classification system of ‘large city group-small city group’ (based on the population size classification) to reveal the spatial and temporal differences in the evolution of PM2.5 concentrations in cities of different sizes. The results of the study can provide scientific basis for the environmental protection and high-quality development of China’s three major urban agglomerations and their city clusters.

## 2. Materials and methods

### 2.1. Study area

The Beijing-Tianjin-Hebei (BTH) urban agglomeration (114°03’ ~ 119°51’ E, 36°20’ ~ 42°40’ N) includes 2 municipalities of Beijing and Tianjin, and all 11 prefectural cities of Hebei Province. According to the “Beijing-Tianjin-Hebei Coordinated Development Plan”, it will be built into a leading area for coordinated development reforms, serve as a new engine for innovation-driven economic growth at the national level, function as a demonstration area for improving environmental restoration, and ultimately develop into a world-class mega-city group centred around the capital.

The Yangtze River Delta (YRD) urban agglomeration (117°05’ ~ 123°25’ E, 28°01’ ~ 34°28’ N) includes Shanghai Municipality, 9 cities in Jiangsu Province, 8 cities in Zhejiang Province, and 8 prefectural cities in Anhui Province. According to its development foundation, the country plans it as an important international gateway in Asia-Pacific region, an important modern service industry and advanced manufacturing base of the world, and a leading-levelled world-class mega-city group.

**Fig 1:**
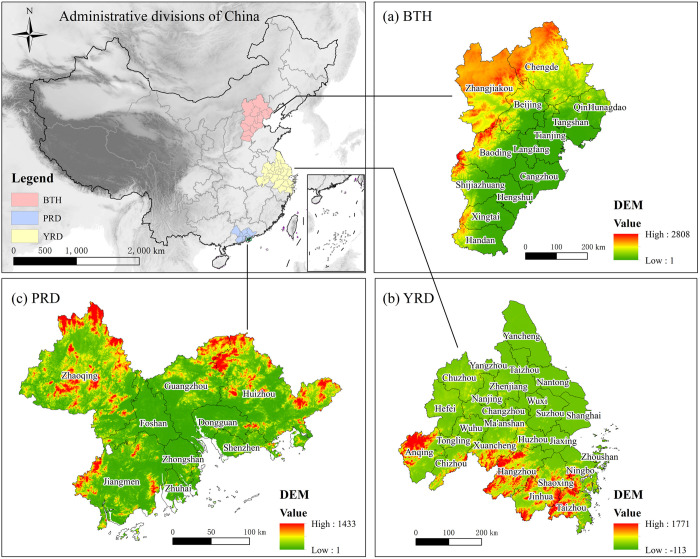
Study area. Note: Republished from [http://bzdt.ch.mnr.gov.cn/] under a CC BY license, with permission from [the Ministry of Natural Resources of the People’s Republic of China], original copyright [2020].

The Pearl River Delta (PRD) urban agglomeration (111°20’ ~ 115°25’ E, 21°27’ ~ 23°57’ N) includes 9 cities in Guangdong Province, forming the Guangdong-Hong Kong-Macao Greater Bay Area. As the forefront of Reform and Opening-up, it has emerged as a significant hub for China’s participations in economic globalization and serves as a national base for the development of scientific and technological innovation. It is also one of the regions with the largest population agglomeration, the strongest innovation abilities and the strongest comprehensive strengths in China. The location of the research area is shown in Fig 1.

### 2.2. Data resources

The original data of PM2.5 in this study is sourced from the grid data of PM2.5 concentration products provided by the Atmospheric Composition Analysis Group of Washington University in St. Louis (https://wustl.box.com/v/ACAG-V5GL04-GWRPM25c0p10). The dataset provides continuity check display and open-source data by administrative region of the annual average PM2.5 concentration in China from 1998 to 2020, with an original spatial resolution of 0.01° × 0.01°. Global and regional PM2.5 concentrations are estimated using information from satellite-, simulation- and monitor-based sources. Aerosol optical depth from multiple satellites (MODIS, VIIRS, MISR, SeaWiFS, and VIIRS) and their respective retrievals (Dark Target, Deep Blue, MAIAC) is combined with simulation (GEOS-Chem) based upon their relative uncertainties as determined using ground-based sun photometer (AERONET) observations to produce geophysical estimates that explain most of the variance in ground-based PM2.5 measurements. A subsequent statistical fusion incorporates additional information from PM2.5 measurements [38], which meet the application research needs of air pollution in China. In addition, ArcGIS software was used to calculate the average PM2.5 concentration of the three urban agglomerations from 2005 to 2020. The specific process is as follows: with ArcGIS software, the raster data of China is cropped and summarised by city (based on the administrative division of 2020), and then the annual average PM2.5 concentration distributions (unit: μg/m^3^) of each city in the three urban agglomerations from 2000 to 2020 are selected.

In the verification of the relationship between the driving factors of PM2.5 concentration evolution in the three urban agglomerations, topographic relief, temperature, precipitation, and vegetation coverage are selected as 4 natural geographical factors. The data of topographic relief and vegetation coverage are derived from the Resource and Environmental Science and Data Centre of the Chinese Academy of Sciences (https://www.resdc.cn); the temperature and precipitation data are derived from the Chinese National Meteorological Science Data Centre (https://data.cma.cn). Eleven socio-economic factors are selected for analysis, including economic scale, population, industrial scale, urban expansion, industrial structure, environmental protection, technological progress, energy efficiency, pollutant emissions, urban open space ratio, and urbanisation ratio. The data mainly comes from the “China City Statistical Yearbook”, “China Construction Statistical Yearbook” and statistical bulletins published by local governments; the urban expansion is measured by the nighttime light index, whose data acquisition refers to the existing practices [39] to obtain the required time series DMSP-OLS data (See Table 1 for details).

**Table 1:** Data sources and descriptions.

Data name	Data description	Data type	Data source
PM2.5 Concentration product	Annual average PM2.5 Concentration	Raster data	Atmospheric Composition Analysis Group of Washington University in St. Louis
RDLS (Relief Degree of Land Surface)	Relief amplitude	Raster data	Resource and Environment Science and Data Center, Chinese Academy of Sciences
Temperature	Annual average temperature	Raster data	Chinese National Meteorological Science Data Centre
Precipitation	Annual average precipitation	Raster data	Chinese National Meteorological Science Data Centre
NDVI(Normalized Difference Vegetation Index)	Vegetation coverage	Raster data	Resource and Environment Science and Data Center, Chinese Academy of Sciences
GDP(Gross Domestic Product)	Scale of economy	Panel data	China City Statistical Yearbook, Statistical Bulletins of Local Government
Permanent population	Scale of population	Panel data	China City Statistical Yearbook, Statistical Bulletins of Local Government
Gross output value of industrial enterprises above designated size	Scale of industrial production	Panel data	China City Statistical Yearbook, Statistical Bulletins of Local Governments
Nighttime light	Urban expansion	Raster data	The Earth Observation Group
Advanced industrial structure index	Industrial structure	Panel data	China City Statistical Yearbook, Statistical Bulletins of Local Governments
Environmental protection investment	Environmental protection efforts	Panel data	China City Statistical Yearbook, Statistical Bulletins of Local Governments
Patent authorisation	Progress of technology	Panel data	China City Statistical Yearbook, Statistical Bulletins of Local Governments
Energy efficiency	Energy consumption	Panel data	China City Statistical Yearbook, Statistical Bulletins of Local Governments
Industrial smoke and dust emission	Pollution emission	Panel data	China City Statistical Yearbook, Statistical Bulletins of Local Governments
Green coverage data of urban areas	Urban Greening rate	Panel data	China City Statistical Yearbook, Statistical Bulletins of Local Governments
Proportion of urban permanent population	Urbanization ratio	Panel data	China City Statistical Yearbook, Statistical Bulletins of Local Governments
Administrative division	Administrative boundaries	Vector data	Resource and Environment Science and Data Center, Chinese Academy of Sciences

### 2.3. Methods

#### 2.3.1. Methods related to spatio-temporal analyses.

(1) Interval classification method. This method divides data into several groups at certain intervals or widths, each group containing a specific range of data. It is suitable for situations where the data is relatively concentrated and naturally distributed throughout the entire range, forming a good integration with software such as ArcGIS. Therefore, it is used to classify the spatial data of PM2.5 in the three urban agglomerations and to analyse their spatial pattern characteristics.
(2) ESDA analysis method. This method is chosen to analyse the spatial autocorrelation and agglomeration of PM2.5 in the three urban agglomerations, taking into account the continuity and concentration of spatial data. Among them, global spatial autocorrelation is reflected by Moran’s I, and local spatial autocorrelation is visualized through the Local Indicators of Spatial Association (LISA) graph drawn by GeoDa software. Thus, the similarity of PM2.5 among the three adjacent or neighbouring urban agglomerations in space can be observed. The formula is as follows [40,41]:

$$I=\frac{\sum_{i=1}^{n}\sum_{j=1}^{n}W_{ij}(Y_{i}-\overset{―}{Y})(Y_{j}-\overset{―}{Y})}{S^{2}\sum_{i=1}^{n}\sum_{j=1}^{n}W_{ij}}$$ (1)

Where, I is Moran’s index; $Y_{i}$ and $Y_{j}$ are the PM2.5 concentration observations of the i-th and j-th cities, $S^{2}$ is the variance of Y, and $w_{ij}$ is the spatial weight matrix. In this study, the adjacency matrix is chosen to calculate the global Moran’s I. The I takes values between [−1, 1]. −1< I<0 indicates that the attribute values of the evaluation object are spatially negatively correlated, I=0 indicates that the attribute values of the evaluation object are not correlated with each other, and 0< I<1 indicates that the attribute values of the evaluation object are spatially positively correlated.

By combining the above two methods, the characteristics of the pattern of PM2.5 concentration evolution can be revealed. As shown in the following figure.

#### 2.3.2. The geodetector.

The geodetector is a powerful tool for the exploratory analysis of spatial data, which can fully explore the process and mechanism behind spatial differentiation. This study takes the average PM2.5 concentration of each city in China’s three major urban agglomerations at different years as the dependent variable and selects 4 natural and 11 socio-economic factors as independent variables. Using the factor detection tool in the geodetector to detect the driving effect of these 15 indicator factors on the spatial differentiation of PM2.5 concentration in the three urban agglomerations, the degree of influence of each factor on the spatial disparities of factors can be indicated, and the relative importance of the 15 factors on the disparities of PM2.5 concentration can be compared. Factor detection mainly relies on the q-statistic values to measure the explanatory power of the 15 indicator factors on the spatial disparities of PM2.5 concentration. The larger the q value, the stronger the explanatory power of the factor for the spatial differentiation of PM2.5 concentration [42,43]. The formula is as follows:


$$q=1-\frac{\sum_{h=1}^{L}N_{h}\delta_{h}^{2}}{{N\delta }^{2}}$$ (2)

Where, L is the independent variable for stratification; $N_{h}$ is the number of units in layer h; N is the overall number of units in the study area; $\delta_{h}^{2}$ and$\;\delta^{2}$ are the variances of layer h and the entire region respectively.

#### 2.3.3. GWR model.

GWR (geographically weighted regression) is a method proposed by Brunsdon et al. in 1996 [44]. This model incorporates spatial heterogeneity and heterogeneity into the regression. It introduces the influence factors of different regions to estimate, which can effectively capture the non-stationary influence of each factor on PM2.5 pollution and describe the characteristics of the spatial variation of the variable relationship. In this study, the GWR model is used to detect the characteristics of the spatial disparities’ factors of PM2.5 concentration in local space. The formula is as follows [44]:


$$\begin{array}{c} Y_{i}=\beta_{0}\left(u_{i},v_{i}\right)+\sum {\mathrm{\backslash nolimits}}_{k=1}^{p}\beta_{k}\left(u_{i},v_{i}\right)X_{ik}+\varepsilon_{i} \end{array}$$ (3)

Where, i is the number of observation units; $Y_{i}$ is the observed PM2.5 concentration value of the i-th city; $\left(u_{i},\;v_{i}\right)$ is the geographic centre coordinate of the observation of the n-th sample; The parameter β is a function of $u_{i}$ and $v_{i}$, meaning that the estimated parameter β for any specific spatial location is obtained through local estimation and varies with different spatial geographic locations; $X_{ik}\;(k=1,2,...,6)$ is the independent variable explanatory value of i city; $\beta_{k}\left(u_{i},v_{i}\right)$ is the regression parameter of the k-th variable of the city i; $\beta_{0}\left(u_{i},v_{i}\right)$ and $\varepsilon_{i}$ are the intercept term and random error term of the city i respectively.

## 3. Analyses of spatio-temporal evolution of PM2.5 concentration in the three urban agglomerations

### 3.1. The concentration of PM2.5 shows an overall trend of fluctuating decline

**Fig 2:**
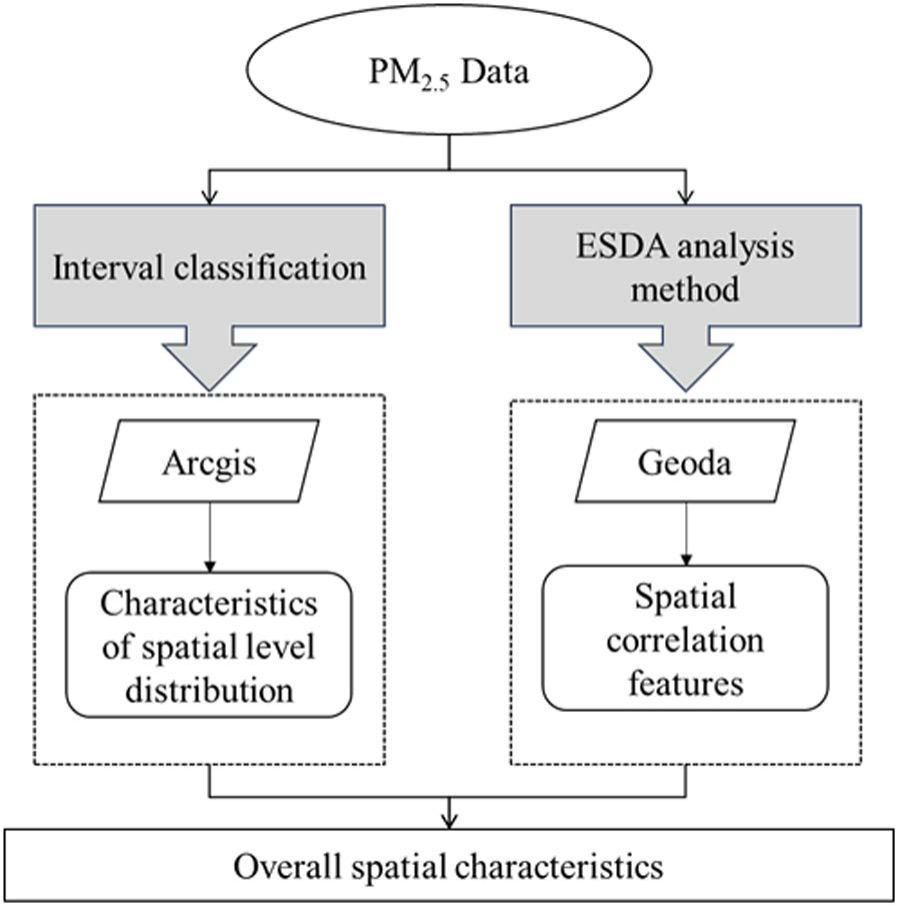
Demonstration of methods for spatial characteristics of PM2.5 concentration.

**Fig 3:**
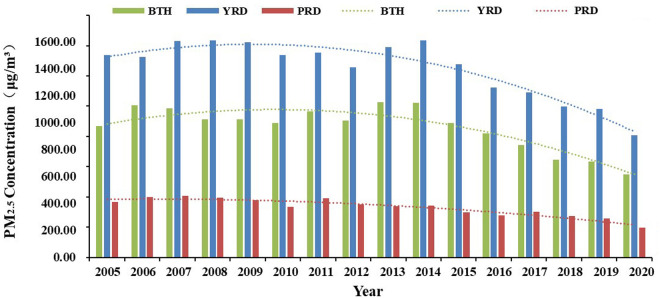
Temporal evolution of PM2.5 concentration in China’s three major urban agglomerations from 2005 to 2020.

The variation range of PM2.5 concentration in the three urban agglomerations is significantly different, and the average annual concentration from 2005 to 2020 shows a fluctuating downward trend (Fig 2), (Fig 3).

The concentration of PM2.5 in the Beijing-Tianjin-Hebei (BTH) region rose to 1004.71 μg/m^3^ in 2006, subsequently decreased to 887.33 μg/m^3^ in 2006–2010, increased again to 1024.78 μg/m^3^ in 2010–2013, and then continued to decline. Notably, PM2.5 pollution was particularly severe in the region during 2006 and 2013, primarily due to emissions from heavy industry, agricultural practices, animal husbandry, and traffic-related emissions [45,46]. Furthermore, the region’s geography, with the Taihang Mountains to the west, impeded the dispersion of pollutants, trapping haze in the area. The concentration of PM2.5 in the Yangtze River Delta (YRD) exhibited a notable increase, rising to 1434.81 μg/m^3^ from 2005 to 2008. This was followed by a decrease to 1255.96 μg/m^3^ from 2008 to 2012, after which it increased again to 1436.39 μg/m^3^ from 2012 to 2014, before experiencing a subsequent decline. The years 2008 and 2014 were characterized by severe PM2.5 pollution levels in the YRD. This phenomenon can be attributed to excessive investment in primary emission control measures in major cities, which failed to address the problem of secondary pollution. The increase and expansion of haze led to an increase in the annual average concentration of PM2.5 [47]. The decrease of PM2.5 levels in the YRD region in 2012 was mainly the result of efforts aimed at energy conservation, emission reduction, air quality management and industrial restructuring [48]. The concentration of PM2.5 in the Pearl River Delta (PRD) region exhibited significant fluctuations between 2005 and 2011. Specifically, the value peaked at 405.02 μg/m^3^ in 2007, subsequently decreased to 336.23 μg/m^3^ from 2007 to 2010, increased again to 392.24 μg/m^3^ from 2010 to 2011, and then experienced a decline. The average annual concentration saw increases in 2007 and 2011, which can be attributed to heightened anthropogenic emissions and changes in meteorological conditions at that time [49]. Regarding meteorological factors, the humid and less windy conditions prevalent in PRD hinder the diffusion and dilution of pollutants, thereby facilitating the accumulation of PM2.5 in the atmosphere. In summary, a common characteristic observed in the temporal patterns of PM2.5 across the three urban agglomerations is a general decreasing trend in fluctuations, suggesting an improvement in air quality to some extent. The differential characteristics of PM2.5 concentrations primarily vary at different time points, with the overall ranking of PM2.5 concentration is: YRD > BTH > PRD.

**Fig 4:**
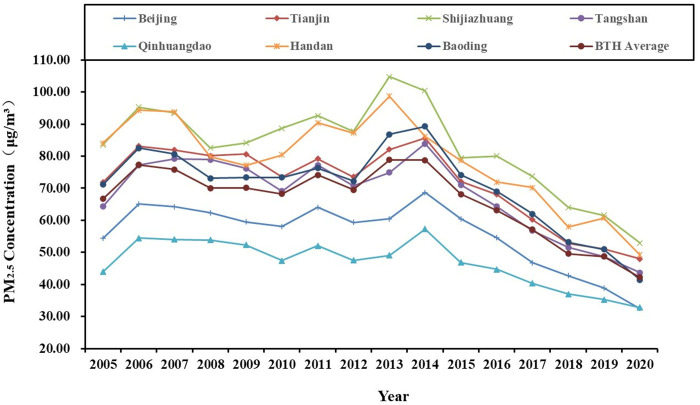
Temporal evolution the annual average PM2.5 concentration of BTH’s “larger city group” from 2005 to 2020.

**Fig 5:**
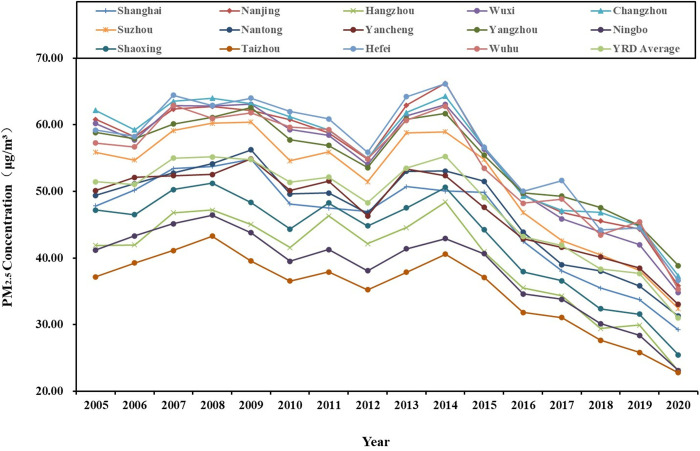
Temporal evolution the annual average PM_2.5_ concentration of YRD’s “larger city group” from 2005 to 2020.

**Fig 6:**
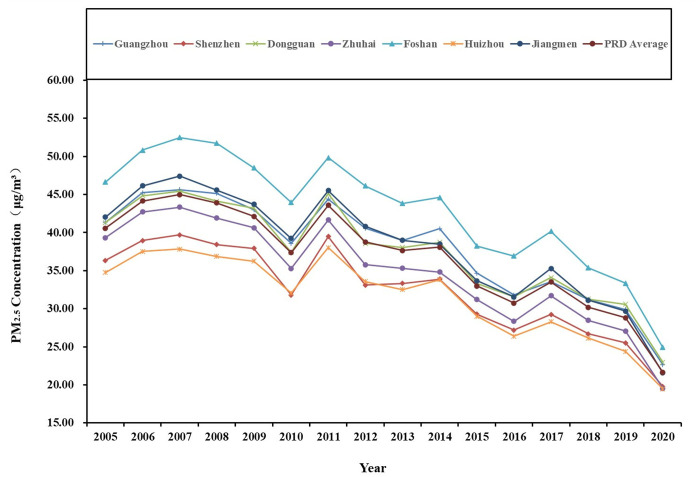
Temporal evolution the annual average PM2.5 concentration of PRD’s “larger city group” from 2005 to 2020.

According to the relevant documents issued by the State Council of China (Notice on Adjusting the Standardisation of Urban Scale Classification, 〔2014〕51), Chinese cities categorized based on resident population size into the following classifications: super-large cities (population exceeding 10 million), mega-large cities (5−10 million), large cities (1−5 million), medium cities (500 thousand – 1 million), and small cities (less than 500 thousand) according to the size of resident population. Therefore, this study classifies the super-large cities, mega-large cities, and large cities as “larger city group”, and the other cities as “smaller city group” (Table 2), then analysing the temporal evolution characteristics of the annual average PM2.5 concentration in the two groups (Figs 4–6).

**Table 2:** “Larger city groups” in China ‘s three major urban agglomerations.

urban agglomeration	super-large cities	mega-large cities	large cities	amounts of cities in “larger city group”
BTH	Beijig,Tianjn	–	Shijiazhuang, Tangshan, Qinhuangdao, Handan, Baoding	7
YRD	Shanai	Nanji, Hangu	Wuxi, Suzhou, Changzhou, Nantong, Yancheng, Yangzhou, Ningbo, Shaoxing, Taizhou, Hefei, Wuhu	14
PRD	Shenn, Dongn	Guangzhou	Zhuhai, Foshan, Huizhou, Jiangmen	7

BTH’s “larger city group”: The data indicates a fluctuating downward trend in values. The value of Beijing among super-large cities is lower than the average level of urban agglomerations, mainly because of relocation of heavy industries from China’s political centre on a large scale as well as the ongoing transition towards a high-end industrial structure. Additionally, improvements in air quality control have yielded positive results. Among the five large cities, only Qinhuangdao exhibits ae value below than the average level of BTH, which can be explained by its coastal location that facilitates air purification through sea breezes. It also shows that the other 5 cities are the primary contributors to haze pollution in urban agglomerations.

YRD’s “larger city group”: The data indicates a fluctuating downward trend in values. The value of super-large city Shanghai hovers around the YRD average level. The value of Hangzhou in mega-large cities falls below than the average level of YRD; among the 11 large cities, only Wuxi, Shaoxing and Taizhou exhibit values below the average level of YRD. These 4 cities are all major ones in eastern China for light industry, commerce, and tourism, and they are characterized by relatively low levels of pollution. This suggests that they are more environmentally sustainable, contributing to the maintenance of good air quality. Conversely, the data implies that the other 9 cities are the primary contributors to haze pollution of urban agglomerations.

PRD’s “larger city group”: The data indicates a fluctuating downward trend in values. The value of super-large city Shenzhen is lower than the average level of PRD. This discrepancy can be attributed to its role as an innovation and financial centre in southern China, where the overall economic operation is environmentally friendly. Among the 4 large cities, only Zhuhai and Huizhou exhibit values lower than the PRD average level, primarily due to their long coastlines and vast hilly forests, which contribute to improved air quality. The value of mega-large city Guangzhou is much higher than the average level of PRD, indicating that it, along with the other 3 large cities, plays a substantial role in the haze pollution affecting urban agglomerations.

In summary, a common feature of the temporal pattern changes of PM2.5 among the larger city group of the three city clusters isa fluctuating and decreasing trend in overall concentration levels, indicating a degree of improvement in air quality. The distinct characteristics of these clusters are mainly shown in the average concentration ranking: BTH > YRD > PRD.

### 3.2. The spatial distribution of PM2.5 concentration shows significant differences

**Fig 7:**
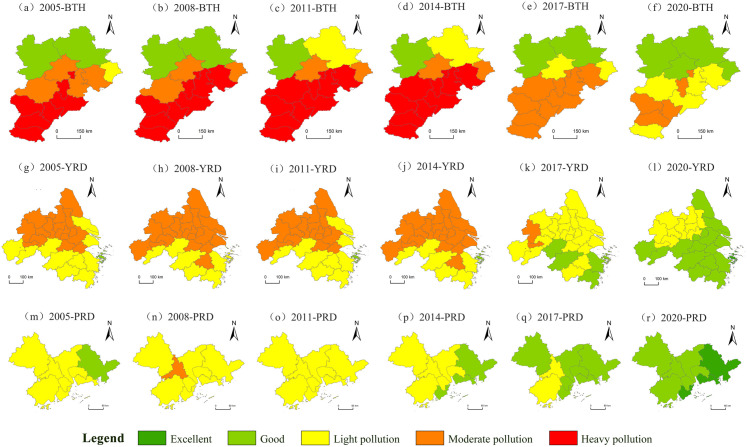
Spatial distribution of air quality levels in China’s three major urban agglomerations from 2005 to 2020. Note: Republished from [http://bzdt.ch.mnr.gov.cn/] under a CC BY license, with permission from [the Ministry of Natural Resources of the People’s Republic of China], original copyright [2020].

In this study, the annual average concentration of PM2.5 (μg/m3) for 6 years (2005, 2008, 2011, 2014, 2017 and 2020) within the study period was evenly selected. The data were visualized using ArcGIS software. According to the World Health Organization (WHO) and China’s “Ambient Air Quality Standards (GB 3095-2012)”, the air quality is categorized into “excellent”, “good”, “mildly polluted”, “moderately polluted” and “severely polluted” levels to derive the spatial distribution and evolution trends of PM2.5 concentration in the three urban agglomerations. The specific division is as follows (Table 3 and Fig 7).

**Table 3:** Air quality classification based on PM2.5 concentration.

serial number	air quality level	PM2.5 concentration interval
1	Excellent	[0, 20]
2	Good	(20, 35]
3	mildly polluted	(35, 50]
4	moderately polluted	(50, 75]
5	severely polluted	(75, +∞)

The PM2.5 concentration of BTH shows a spatial distribution characterized by higher levels in the southern areas. From 2005 to 2008, the air quality levels in 2 cities in the BTH were rated as “good”, the count of cities with “mildly polluted” level decreased by 1, the count of cities with “moderately polluted” level decreased by 1, while the count of cities classified as “severely polluted” level remained unchanged. In these three years, the air quality of some cities has been improved, and yet the air quality of some cities was still poor. From 2008 to 2014, 1 city’s air quality level was “good”, the number of cities with “mildly polluted” level decreased by 1, and the number of cities with “moderately polluted” level decreased by 1. From 2014 to 2017, the air quality level of 2 cities was “good”, the number of cities with “mildly polluted” level decreased by 1, the number of cities with “moderately polluted” level increased to 5, and the cities with “severely polluted level disappeared. From 2017 to 2020, the air quality level of 4 cities was “good”, the number of cities with “mildly polluted” level increased by 3, the number of cities with “severely polluted” level decreased to 4. The number of “polluted” cities decreased by 18.18%, indicating that the air quality of BTH cities in these 6 years has generally improved. The main reason for the high PM2.5 concentration in the south is that the industrial structure needs to be optimised. Numerous projects with high energy consumption, significant pollution, and resource dependence contribute to substantial pollutant emissions. In addition, natural factors play a role [50]. the region’s topography, which is higher in the west and lower in the east, combined with the susceptibility to El Niño events, results in weak cold air activity and frequent occurrences of humidity inversion, thereby impairing the dispersion conditions for PM2.5.

The PM2.5 concentration of YRD shows a spatial distribution characterized by higher levels in the northwest areas. From 2005 to 2014, 1 city in YRD having a “good” air quality rating, the number of cities with “mildly polluted” level decreased by 5, and the number of cities with “moderately polluted” level increased by 5. The increase in polluted cities indicates that the improvement of air quality in some cities in the YRD has not been effective in these 10 years. From 2014 to 2017, the air quality of 4 cities improved to “good” level, the number of cities with “mildly polluted” level increased by 13, and the number of cities with “moderately polluted” level decreased significantly to 2. The areas with superior air quality began to be concentrated in the southeast. From 2017 to 2020, the air quality grade of one city was “excellent”, the number of cities with “good” level increased by 10, the number of cities with “mildly polluted” level decreased to 10, and the cities with “moderately polluted” and “severe polluted” levels disappeared. It shows that the air quality of YRD cities have been significantly improved in these three years. The concentration of PM2.5 in the northwest is high, primarily due to the necessity for optimization of the industrial structure, as well as geographical factors [50].

The PM2.5 concentration of PRD shows an overall spatial distribution characteristic of “decreasing in two levels from west to east.”. From 2005 to 2008, the air quality level of 1 city was “good”, the number of cities with “mildly polluted” level decreased by 1, the number of cities with “moderately polluted” level decreased by 1, and 8 cities were in the “mildly polluted” level. From 2008 to 2014, the air quality of the three cities was “good”, and the number of cities with “mildly polluted” level decreased by 2. In 2011 and 2014, there were only 2 levels among PRD cities; from 2014 to 2020, the air quality improvement effect of PRD cities was remarkable. In general, the air quality of PRD is better than that of BTH and YRD.

### 3.3. The evolution of PM2.5 concentration patterns exhibits significant spatial clustering characteristics

With the help of GeoDa software, the global Moran’s index, Z-value and P-value (Table 4) are calculated to reveal the spatial correlation characteristics of PM2.5 annual average concentration in the three major urban agglomerations in China. It can be seen that the PM2.5 concentration of the three urban agglomerations shows distinct spatial autocorrelation after 16a of evolution, but the overall Moran’s index is significantly greater than 0, showing a positive autocorrelation agglomeration distribution. The evolution of the Moran’s indexes for BTH, YRD, and PRD is significant fluctuations. Among them, BTH and PRD show fluctuating downward trends, while YRD shows a fluctuating upward trend. This indicates that cities with higher PM2.5 concentrations in BTH and PRD have positive impacts on the increase of PM2.5 concentrations in neighbouring cities, while such impacts in YRD appear to be weaker.

**Table 4:** Moran’s index of global autocorrelation of PM2.5 concentration in three major urban agglomerations in China from 2005 to 2020.

region	year	Moran’s I	Z statistic	P-value	year	Moran’s I	Z statistic	P-value
BTH	2005	0.606	3.6089	0.002	2014	0.463	2.8050	0.012
	2008	0.414	2.8576	0.010	2017	0.577	3.5175	0.004
	2011	0.552	3.5614	0.003	2020	0.528	3.1134	0.005
YRD	2005	0.417	3.7075	0.002	2014	0396	3.5844	0.001
	2008	0.315	2.9089	0.007	2017	0.483	4.1599	0.001
	2011	0.381	3.5654	0.002	2020	0.545	4.7313	0.001
PRD	2005	0.286	1.7984	0.051	2014	0.316	2.0598	0.040
	2008	0.372	2.3112	0.020	2017	0.329	2.0722	0.021
	2011	0.278	1.6310	0.048	2020	0.285	1.7842	0.056

**Fig 8:**
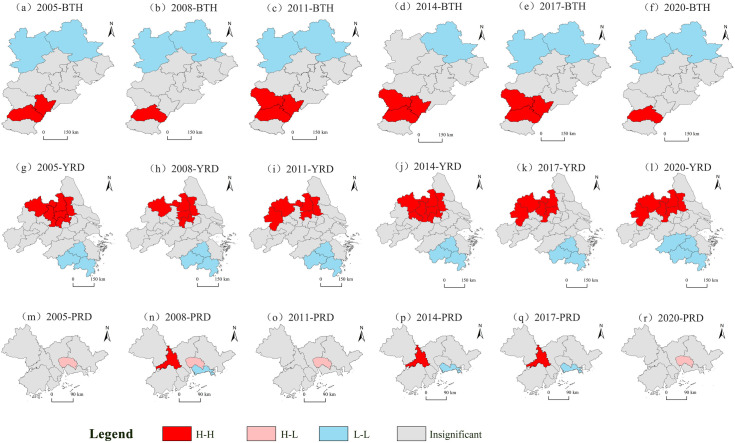
The LISA diagrams of PM2.5 concentration of China’s three major urban agglomerations from 2005 to 2020. Note: Republished from [http://bzdt.ch.mnr.gov.cn/] under a CC BY license, with permission from [the Ministry of Natural Resources of the People’s Republic of China], original copyright [2020].

In general, the agglomeration areas of H-H and L-L in BTH have minimal changes, in which Hengshui has demonstrated an improvement in air quality, whereas Shijiazhuang has experienced a deterioration (Fig 8). From the evolution trend, the extent of pollution levels is contracting. The accumulation of L-L is mainly situated in the north, where the air quality of these cities has remained stable at a good level for a long time. H-H is mainly distributed in the south, represented by Xingtai, Hengshui and Shijiazhuang. The air quality of these cities has been stable at the level of pollution for a long time. The agglomeration areas of H-H and L-L in YRD have minimal changes over time, among which Wuxi has improved its air quality, while Hefei has deteriorated. From the evolution trend, the scope of the pollution levels has not undergone significant alterations. L-L is mainly located in the southeast, indicating that the air quality of these cities has been stable at a good level for a long time. H-H is mainly distributed in the northwest of the YRD, represented by Yangzhou, Chuzhou and Hefei. The air quality of these cities has been stable at the level of pollution for a long time. The types of agglomeration areas of PRD vary greatly, among which the air quality in Shenzhen has made better, while that in Zhongshan has deteriorated. From the evolution trend, the range of H-L has not changed. L-L is mainly located in the southeast, indicating that the air quality of these cities has been stable at a good level for a long time. H-H is mainly distributed in the west, represented by Foshan, and the air quality of these cities has been stable at the level of pollution for a long time.

## 4. Quantitative attribution analysis of spatial pattern evolution

Existing studies have shown that the formation of PM2.5 pollution and the factors influencing the evolution of its spatial pattern include two aspects: natural geographical factors and socio-economic factors [40,50,51]. This study combines relevant literature to establish a driving force index system that elucidates the spatial differentiation of PM2.5 concentrations across the three urban agglomerations (Table 5) [48,52,53,54–57]. The geodetector methodology, which is adept at identifying spatial differentiation and interactions, is employed to measure the influence of different driving factors on the evolution of PM2.5 concentration pattern within the three urban agglomerations. Between 2005 and 2020, the driving factors with large variation were identified, and the top 8 driving factors were selected to characterise the spatial effects of PM2.5 concentrations.

**Table 5:** The driving force index system of spatial disparities of PM2.5 concentration of China’s three major urban agglomerations.

driving type	factor	measurement	serial	unit
natural geographical	topographic relief	RDLS	*X1*	m
	temperature	mean annual temperature	*X2*	°C
	precipitation	mean annual precipitation	*X3*	mm
	vegetation coverage	NDVI	*X4*	–
socio- economic	economic scale	GDP	*X5*	10^4^ RMB
	population	number of permanent residents	*X6*	10^4^ RMB
	industrial scale	industrial output value above certain scale	*X7*	10^4^ RMB
	urban expansion	nighttime light index	*X8*	–
	industrial structure	index of industrial structure upgrading	*X9*	–
	environmental protection	Environmental protection expenditure÷total government public expenditure	*X10*	%
	technology progress	number of patent authorisations	*X11*	–
	energy efficiency	GDP÷industrial power consumption	*X12*	%
	pollutant emissions	industrial soot emissions	*X13*	t
	urban open space ratio	urban green coverage ratio	*X14*	%
	urbanisation	urbanisation ratio	*X15*	%

### 4.1. The evolution of PM2.5 concentration patterns is influenced by the combined effects of natural factors and socioeconomic factors

From detection results of the driving factors of spatial disparities of PM2.5 concentration in the three urban agglomerations (Table 6), it can be seen that both natural geographical and socio-economic factors interact to exhibit characteristics of spatial disparity. In 2005, the factors contributing significantly to the spatial disparities in PM2.5 concentration included temperature (X2), precipitation (X3), pollutant emissions (X13), population (X6), urbanisation (X15), industrial structure (X9), energy efficiency (X12), and technology progress (X11) were more pronounced. In contrast, the influences of economic scale (X12) and industrial scale (X7) were lesser. In 2017, factors such as temperature (X2), precipitation (X3), energy efficiency (X12), urbanisation (X15), industrial structure (X9), vegetation coverage (X4), topographic relief (X1), and technological progress (X11) contributed significantly to the spatial differentiation of PM2.5 concentrations in the three major urban agglomerations while economic scale (X7) and urban open space ratio (X14) had a smaller contribution. In 2020, factors such as temperature (X2), precipitation (X3), urbanisation (X15), vegetation coverage (X4), urban expansion (X8), population (X6), technology progress (X11) and environmental protection (X10) had a significant contribution to the spatial differentiation of PM2.5 concentrations in the three major urban agglomerations, while urban open space ratio (X14) and economic scale (X7) had a smaller contribution

**Table 6:** Detection results of driving factors (q value) of spatial disparities of PM2.5 concentration in China’s three major urban agglomerations from 2005 to 2020.

driving type	factor serial	q value					
2005	rank	2017	rank	2020	rank
**natural** **geographical**	X1	0.096***	9	0.106***	7	0.100***	9
	X2	0.509***	1	0.729***	1	0.751***	1
	X3	0.459***	2	0.460***	2	0.380***	2
	X4	0.082***	10	0.116***	6	0.152***	4
socio- economic	X5	0.032***	14	0.039***	12	0.044***	13
	X6	0.177***	4	0.070***	10	0.121***	6
	X7	0.025***	15	0.029***	14	0.029***	15
	X8	0.051***	12	0.065***	11	0.125***	5
	X9	0.154***	6	0.128***	5	0.032***	14
	X10	0.037***	13	0.030***	13	0.102***	8
	X11	0.103***	8	0.086***	8	0.111***	7
	X12	0.136***	7	0.140***	3	0.065***	12
	X13	0.360***	3	0.071***	9	0.081***	10
	X14	0.070***	11	0.021***	15	0.077***	11
	X15	0.166***	5	0.133***	4	0.159***	3

The ranking results of q statistics show that temperature and precipitation, as natural factors, have the highest q values, explaining over 35% of the spatial differentiation of PM2.5 concentrations in the three major urban agglomerations. Vegetation coverage, population, industrial structure, urbanisation and technology progress explain between 10% and 20% of the spatial differentiation of PM2.5 concentrations, categorizing them as secondary factors. Conversely, other factors consistently explain less than 10% of the spatial differentiation, suggesting a relatively minor yet discernible impact. In 2005 and 2020, natural geographical factors had a greater influence than socio-economic factors, with the former having a more pronounced impact on the changes in PM2.5 pollution levels in the three urban agglomerations.

### 4.2. The interaction of driving factors is manifested as a two-factor enhancement or a nonlinear enhancement effect

**Fig 9:**
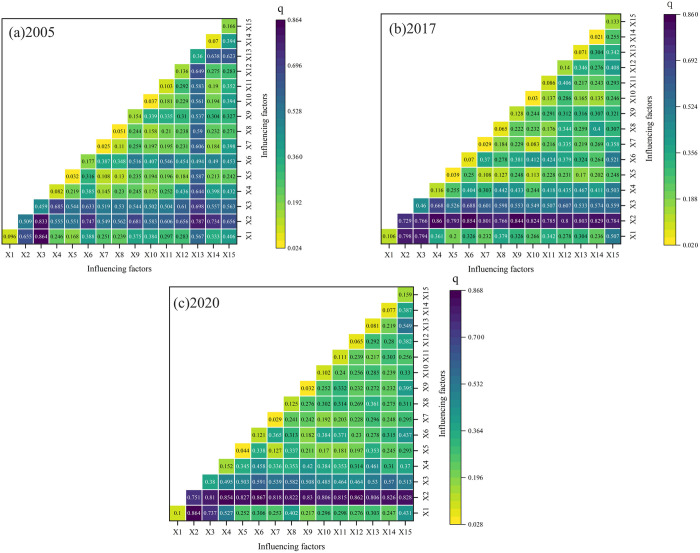
Detection results of interaction among influencing factors.

Interaction detection aims to assess whether the interaction between factors will increase or decrease the explanatory power of the dependent variable, or whether the effects of these factors on the dependent variable are independent of each other. In this study, the interaction detection of geodetector was utilized to assess the explanatory power of the mutual coupling and mutual feedback relationships among factors contributing to the spatial disparities of PM2.5 concentration across the three urban agglomerations were obtained. The results show two types of “dual-factor enhancement” or “nonlinear enhancement” (Fig 9). In 2005, the top 4 combinations were X1∩X3 (0.864), X2∩X3 (0.833), X2∩X13 (0.787), X2∩X6 (0.747); in 2017, the top 4 combinations were X2∩X4 (0.860), X2∩X6 (0.854), X2∩X9 (0.844), X2∩X14 (0.829); in 2020, the top 4 combinations were X2∩X6 (0.867), X1∩X2 (0.864), X2∩X12 (0.862), X2∩X4 (0.854). The explanatory power of the interaction between variable X2 and other factors is predominantly greater than the influence of X2’s own disparity detection, which aligns with its significant role in disparity detection. The “dual-factor enhancement” type in 2017 and 2020 includes X2∩X6 and X2∩X4. In the disparity detection, X2 has the strongest explanatory power; in the dual-factor enhancement type, the strongest combination of explanatory power is X2∩X6, with a significant improvement in explanatory power. In summary, when X2 respectively interacts with other factors, the explanatory power of spatial disparities of PM2.5 concentration will be improved, so as to enhance spatial heterogeneity. This also indicates that X2 plays a key role in the evolution of PM2.5 concentration distribution, because under high-temperature conditions or when the temperature rises, the movement of air molecules accelerates, intensifying the atmospheric convection effect, which is conducive to the diffusion and dilution of pollutants, thereby constituting a natural condition conducive to the dissipation of PM2.5 pollution.

### 4.3. The driving forces behind the evolution of PM2.5 concentration patterns exhibit significant spatial heterogeneity

**Fig 10:**
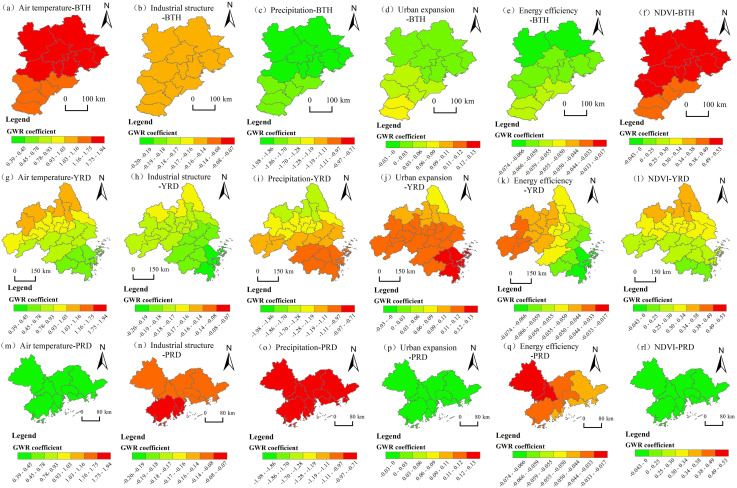
Regression coefficient distributions of driving factors of PM2.5 concentration in China’s three major urban agglomerations in 2005. Note: Republished from [http://bzdt.ch.mnr.gov.cn/] under a CC BY license, with permission from [the Ministry of Natural Resources of the People’s Republic of China], original copyright [2020].

**Fig 11:**
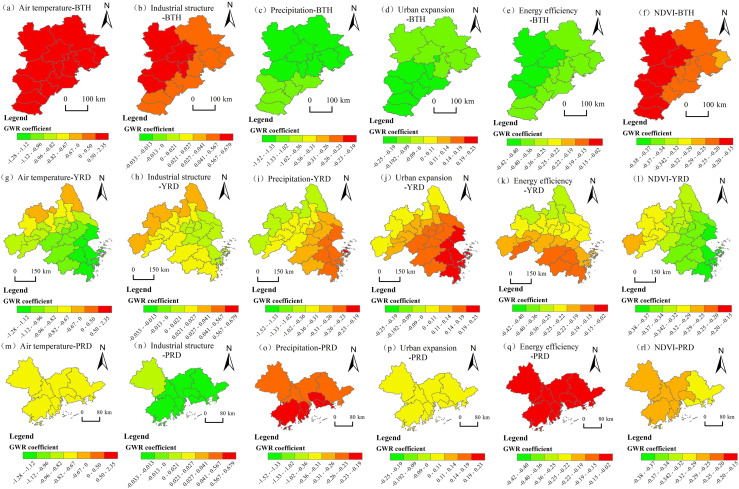
Regression coefficient distributions of driving factors of PM2.5 concentration in China’s three major urban agglomerations in 2020. Note: Republished from [http://bzdt.ch.mnr.gov.cn/] under a CC BY license, with permission from [the Ministry of Natural Resources of the People’s Republic of China], original copyright [2020].

The top 6 driving factors with significant differences in the spatial disparity detection results of PM2.5 concentration in the three urban agglomerations in 2005 and 2020 are: *X2* > *X9* > *X3* > *X8* > *X12* > *X4*. In this study, these 6 factors, with the largest changes in the explanatory power of the differentiation detection in the geographical detector at the beginning and end of the study period, are fitted into a geographically weighted regression model. The spatial and temporal differences in the direction and intensity of the influencing factors are analysed through the regression coefficients in the geographically weighted regression (Figs 10,11).

1) In 2005, the coefficient of temperature (X2) was basically positive, which enhanced the PM2.5 pollution. The coefficients of industrial structure (X9), precipitation (X3), urban expansion (X8), energy efficiency (X12), and vegetation coverage (X4) are basically negative, which had restraining effects on PM2.5 pollution. In 2020, precipitation (X3), energy efficiency (X12), and vegetation coverage (X4) had inhibitory effects on PM2.5 pollution. Temperature (X2) has an enhanced effect on PM2.5 pollution in BTH, a weak enhancement effects on YRD, and an inhibitory effect on PM2.5 pollution in PRD. The industrial structure (X9) had an enhanced effect on PM2.5 pollution in BTH and YRD, and had an inhibitory effect on PM2.5 pollution in PRD. Urban expansion (X8) had an enhanced effect on PM2.5 pollution in YRD and PRD, and had an inhibitory effect on PM2.5 pollution in BTH. In general, the inhibitory effects of the above 6 factors on PM2.5 concentration increased over during the study period in the three urban agglomerations.
2) In terms of natural geographical factors, temperature (X2) exerted a positive inhibitory effect on PM2.5 pollution in the three major urban agglomerations, and the inhibitory effect was more concentrated in YRD; precipitation (X3) generally had a positive inhibitory effect on PM2.5 pollution in the three major urban agglomerations, with a more significant inhibitory effect on PM2.5 pollution in BTH, indicating that precipitation can more effectively promote the reduction of haze pollution; vegetation coverage (X4) also gradually exerted a positive inhibitory effect on PM2.5 pollution in the three major urban agglomerations, and the inhibitory effect was more significant in BTH, showing a concentrated trend. Thus, compared with the other two urban agglomerations, the reduction of PM2.5 pollutants in the BTH region is more significantly influenced by natural factors.
3) In terms of socio-economic factors, the industrial structure (X9) inhibited the aggregation of PM2.5 in PRD, indicating that the PRD region has achieved phased results in the green transformation and development of energy under the background of “ecological civilization construction”. The impact of urban expansion (X8) showed a trend of spreading from PRD to BTH, suggesting that under the support of natural geographical conditions, the cities in BTH are more conducive to enhancing the pollution reduction effect through the expansion of built-up areas than those in PRD, while the effect on BJH is not significant. The regression coefficient of energy efficiency (X12) was generally negative during the research period, indicating that the improvement of energy efficiency in the three major cities had a good inhibitory effect on PM2.5 pollution, especially in the southeast of the BTH region, where the inhibitory effect on PM2.5 pollution was more significant.

## 5. Discussions

### 5.1. Key findings

The environmental problems of urban agglomerations will restrict their high-quality development. Haze pollution in China is particularly serious, and urban agglomerations have become a gathering place for PM2.5 concentrations [58,59]. As complex systems, the urban agglomerations’ development is always accompanied by various environmental issues and governance challenges that warrant serious attention. These factors are also important reasons for the developing gap between China’s three major urban agglomerations and the 5 existing high-quality urban agglomerations in the world.

While conducting the research, it has been found compared with previous studies that most ones focused on PM2.5 concentration’s evolution within individual cities or urban agglomerations, lacking a systematic contrast of the three major urban agglomerations in China [50]. For instance, earlier research often used ground monitoring data to provide simple descriptions of spatiotemporal changes in PM2.5 concentration. In recent years, the increasing application of remote sensing technology and spatial analysis methods has enabled researchers to more comprehensively reveal the spatiotemporal evolution characteristics of PM2.5 in China’s three major urban agglomerations [60]. In addition, existing research on the driving factors focused more on single factors such as industrial emissions and energy consumption, while often overlooking the integrated effects of regional interactions and policy regulation [61–63]. Similarly, new studies have been gradually focusing on spatial spillover effects of PM2.5 pollution across regions, as well as the comprehensive impacts of various industrialisational and urbanisational aspects in different urban agglomerations on PM2.5 evolution [64]. Those provide a more compositive perspective for understanding the spatiotemporal evolution of PM2.5 in China’s three major urban agglomerations, and also a scientific basis for regional air pollution control and policy formulation.

The study of PM2.5 pollution in urban agglomerations is helpful to urban beautification [65], aligning with China’s developmental strategy. Effectively enhancing air quality and improving the level of urban liveable environment is a grand action that requires the participation of the government, enterprises, scholars, and the people. This paper reveals the evolution pattern of PM2.5 pollution and its spatial agglomeration across China’s three major urban agglomerations from a geographical perspective, and clarifies the dominant factors of PM2.5 pollution and their influencing mechanism, providing a scientific basis for the attribution, simulation, early warning, prevention and controlling of air pollution in China’s urban agglomerations, as well as for industrial planning and healthy urban development.

### 5.2. Policy implications

Although PM2.5 pollution levels in the three urban agglomerations have exhibited a fluctuating trend of improvement trend in recent years, certain cities continue to experience adverse effects due to their large population sizes and reliance on traditional production methods. The task of urban beautification is arduous, and efforts toward atmospheric governance require substantial further development. In this regard, the following recommendations are made:

1) Establish cooperation mechanisms for prevention and remediation of PM2.5 pollution beyond urban agglomerations. According to different emission sources, policies referring to local conditions should be made sound; at the same time, the division of labour and coordinated development among the core cities should be enhanced, and an efficient modern development situation of urban agglomerations should be formed.
2) Construct PM2.5 pollution control frameworks with the participation of government, enterprises and citizens. The government should enhance the ecological functions and promote the green transformation through the implementation of environmental regulations. Enterprises are obligated to fulfill their social responsibility by addressing issues related to high energy consumption and high emissions. Additionally, citizens should cultivate environmental awareness and adopt sustainable lifestyles.
3) Coordinating greening and low-carbon development in key regions and areas. The promotion of green and low-carbon practices represents significant trends and objectives in the transformation and enhancement of development. Under the premise of attaching significance to relevant strategies, it is imperative for the nation to bolster cooperation in greening development and to engage actively in international collaboration and competition. This includes the establishment of demonstration areas for green development, the expansion of green industrial supply chains, and the advancement of green technologies, among other initiatives.

### 5.3. Limitations and future research

#### 5.3.1. Limitations.

The research acknowledges certain limitations that could be improved in future studies. First, there are limitations in data sources and quality. Some of the socio-economic indicators (e.g., night light index to measure urban expansion) may not fully capture the details of urbanisation (e.g., land use type, density of transport network), and the difference in statistical dimensions across different years or regions may affect the comparability of the results.

Second, the limitations of model assumptions. GWR assumes local smoothness of spatial relationships, but the actual PM2.5 driving mechanism may show non-smoothness with scale or regional context (e.g., coastal vs. inland), and the model results may be affected by the sensitivity of bandwidth selection, which has not been adequately examined in relation to the underlying mechanisms of spatial heterogeneity.

Finally, the issues of spatio-temporal scale and the generalizability of the findings. The study is based on annual mean analysis, which could not capture the seasonal, monthly or daily patterns of PM2.5 changes (e.g., pollution peaks during the winter heating season), and may mask differences in short-term driving mechanisms. In addition, meteorological factors (e.g., wind speed, inversion layer frequency) are only characterised by annual mean temperature and precipitation, ignoring the triggering effect of short-term extreme weather events (e.g., stationary weather) on pollution accumulation, which may weaken the dynamic influence of natural conditions.

#### 5.3.2. Future research.

On the basis of the findings in this paper, the follow-up studies also need to deeply analyse the main pollution sources of PM2.5 pollution Additionally, these studies should simulate the PM2.5 transmission path and plan the PM2.5 circulation corridor in the region. Furthermore, the control of PM2.5 pollution represents a complex system that necessitates cross-sectoral, interdisciplinary, and inter-organizational collaboration. This collaboration should facilitate research that is multifactorial, multiscale, multidimensional, and utilizes various models. The mechanisms of PM2.5 pollution at all levels are miscellaneous, so accurate research will be more conducive to the classification and guidance of future development policies.

## 6. Conclusions

This paper draws the following major conclusions through empirical research:

1) The spatial pattern of PM2.5 concentration in China’s three major urban agglomerations is mainly as follows: ① The PM2.5 annual average concentration in urban agglomerations and their “larger city groups” shows a fluctuating downward trend, which aligns with the findings of existing studies [50, 66]. ② There is a significant differentiation in PM2.5 concentration levels among the three urban agglomerations, all of which are trending towards optimal levels. ③ The spatial correlation evolution of PM2.5 concentration in urban agglomerations shows positive autocorrelation. ④ The PM2.5 concentration pattern differentiations in urban agglomerations are affected by natural geographical and socio-economic factors, with the impact of natural geographical factors being more pronounced.
2) The disparities of quantitative attribution of PM2.5 concentration pattern across China’s three major urban agglomerations are as follows: ① In terms of time series, YRD > BTH > PRD. ② In terms of spatial evolution, the pattern of BTH represents “high in the south”, the pattern of YRD represents “high in the northwest”, and the pattern of PRD represents “west-east bipolar decreasing”. ③ In terms of spatial correlation, the PM2.5 agglomeration types of BTH and YRD are mainly “high-high” and “low-low”, and the agglomeration types of PRD are “high-high”, “low-low” and “high-low”. The areas experiencing severe pollution in BTH and YRD are gradually shrinking. ④ The factors influencingPM2.5 concentration patterns in the three urban agglomerations exhibit varying degrees of significance. Temperature and vegetation coverage inhibited PM2.5 pollution in BTH and YRD, corroborating findings from existing studies [67 , 68]. Precipitation most significantly inhibited PM2.5 pollution in BTH, aligning with the conclusions from prior research [69]. Industrial structure inhibits the PM2.5 pollution of PRD, consistent with findings from previous studies [70]. The effect of urban expansion on PM2.5 pollution spread from PRD to BTH; energy efficiency demonstrates the most significant inhibitory effect on PM2.5 pollution in the southeast of YRD, further supporting conclusions from existing literature [71].
